# Clinical Reasoning Skills in Psychiatric Education: Scenario and Question Generation with Chat-GPT

**DOI:** 10.1192/j.eurpsy.2025.2380

**Published:** 2025-08-26

**Authors:** B. Ozel, E. Emekli, E. Emekli

**Affiliations:** 1 Department of Psychiatry, Baskent University, Ankara; 2 Department of Radiology, Eskişehir Osmangazi University, Eskişehir, Türkiye

## Abstract

**Introduction:**

Case-based learning holds a crucial place in psychiatric education. Through methods such as written scenarios, video presentations, patient simulations, or observing real patients, students are expected to gain clinical reasoning skills by observing psychiatric cases.

**Objectives:**

This study aims to generate case scenarios using ChatGPT-4 and create multiple-choice questions based on these cases.

**Methods:**

A prompt was developed based on the literature to generate case scenarios for 12 psychiatric diagnoses, along with five related questions for each case (Figure) (Kıyak. Rev esp educ méd 2023; 4(3)). The scenarios and questions were organized into six forms, each containing two cases and ten questions.

**Results:**

A total of 12 psychiatrists, (5.33 ± 1.31 years of practice) evaluated each form in pairs. The results of the case evaluations are presented in Table 1, and the question evaluations in Table 2.

**Table:** 

Table 1	Form 1	Form 2	Form 3	Form 4	Form 5	Form 6	Total
	Schizophrenia/ MDD	Schizophreniform/ AN	Brief PD/ SSD	Panic/ Bipolar I	Dysthymia/ BPD	Conversion/ GAD	
The clinical scenario represents a typical case for the queried disorder.	2/2	2/2	1/2	2/2	2/2	2/2	23/24
The mental status examination findings in the case are appropriate for diagnosis.	2/2	2/2	2/2	2/2	2/2	1/2	23/24
The case scenario is well-written.	2/2	2/1	2/2	2/2	2/2	2/2	23/24
The case is of appropriate difficulty for medical students.	2/2	1/1	2/1	2/2	2/2	1/2	20/24
The case is aimed at measuring clinical reasoning skills.	2/2	2/2	2/1	2/2	1/1	2/2	21/24

**Table:** 

Table 2	The question text is clear.	The question is clinically appropriate.	The question has only one correct answer.	The information provided is sufficient to find the correct answer.	The distractors are plausible.	The question was of appropriate difficulty for medical students.	Factual Recall/ Clinical reasoning	Total by question type
**Diagnosis**	23	23	22	23	23	19	16	149/168
**Treatment**	24	23	12	20	17	15	17	128/168
**Differential diagnosis**	22	17	18	16	19	15	20	127/168
**Prognosis**	23	22	17	21	19	14	14	130/168
**Complications**	21	19	17	19	13	12	14	115/168
**Total by criterion**	113/120	104/120	86/120	99/120	91/120	75/120	81/120

**Conclusions:**

The evaluation confirmed that the case scenarios were typical for the respective disorders and included appropriate mental status examinations for diagnosis (23/24). The cases were deemed suitably challenging for medical students (20/24) and effective in assessing clinical reasoning skills (21/24). However, the questions did not meet certain criteria, such as appropriate difficulty (75/120), the presence of a single correct answer (86/120), and the plausibility of distractors (91/120). It is suggested that further work on the prompt is needed to improve the quality of the questions. While ChatGPT is suitable for generating case scenarios, the questions should be reviewed before use.

**Disclosure of Interest:**

None Declared

